# Serum protein profiling reveals an inflammation signature as a predictor of early breast cancer survival

**DOI:** 10.1186/s13058-024-01812-x

**Published:** 2024-04-09

**Authors:** Peeter Karihtala, Suvi-Katri Leivonen, Ulla Puistola, Elina Urpilainen, Anniina Jääskeläinen, Sirpa Leppä, Arja Jukkola

**Affiliations:** 1 Department of Oncology, Helsinki University Hospital Comprehensive Cancer Center, University of Helsinki, P.O. Box 180, Helsinki, FI-00029 Finland; 2 Department of Oncology and Radiotherapy, Medical Research Center Oulu, Oulu University Hospital and University of Oulu, Oulu, Finland; 3 Applied Tumor Genomics, Research Programs Unit, Medical Faculty, University of Helsinki, Helsinki, Finland; 4 iCAN Digital Precision Cancer Medicine Flagship, Helsinki, Finland; 5 Department of Obstetrics and Gynecology, Medical Research Center, Research Unit of Clinical Medicine, University of Oulu and Oulu University Hospital, Wellbeing Services County of North Ostrobothnia, Oulu, Finland; 6 Department of Oncology, Tampere Cancer Center, Faculty of Medicine and Health Technology, Tampere University Hospital, Tampere University, Tampere, Finland

**Keywords:** Blood, Breast cancer, Proteomics, Prognostic factor, Proximity-extension assay

## Abstract

**Background:**

Breast cancers exhibit considerable heterogeneity in their biology, immunology, and prognosis. Currently, no validated, serum protein-based tools are available to evaluate the prognosis of patients with early breast cancer.

**Methods:**

The study population consisted of 521 early-stage breast cancer patients with a median follow-up of 8.9 years. Additionally, 61 patients with breast fibroadenoma or atypical ductal hyperplasia were included as controls. We used a proximity extension assay to measure the preoperative serum levels of 92 proteins associated with inflammatory and immune response processes. The invasive cancers were randomly split into discovery (*n* = 413) and validation (*n* = 108) cohorts for the statistical analyses.

**Results:**

Using LASSO regression, we identified a nine-protein signature (CCL8, CCL23, CCL28, CSCL10, S100A12, IL10, IL10RB, STAMPB2, and TNFβ) that predicted various survival endpoints more accurately than traditional prognostic factors. In the time-dependent analyses, the prognostic power of the model remained rather stable over time. We also developed and validated a 17-protein model with the potential to differentiate benign breast lesions from malignant lesions (Wilcoxon *p* < 2.2*10^− 16^; AUC 0.94).

**Conclusions:**

Inflammation and immunity-related serum proteins have the potential to rise above the classical prognostic factors of early-stage breast cancer. They may also help to distinguish benign from malignant breast lesions.

**Supplementary Information:**

The online version contains supplementary material available at 10.1186/s13058-024-01812-x.

## Background

Despite the steadily improving prognosis of early-stage breast cancer, 25–30% of patients still succumb to their disease [1–3]. Historically, TNM classification and immunohistochemical stainings, such as estrogen receptor (ER) and Ki-67, have been used for risk stratification and to aid in the optimization of treatment and surveillance intensity. More recently, tissue-based prognostic assays such as MammaPrint and Oncotype DX have been adopted to guide clinical decisions, although logistics and affordability may limit their use [3–6].

Serum proteins could have several advantages as biomarkers over the above-mentioned classical prognostic factors both in early diagnosis and in the risk stratification of early-stage breast cancer patients. First, bloodstream protein expression could offer easily accessible and minimally invasive means of assessing tumor biology [7]. Serum proteomics can also provide information on systemic changes in response to the tumor, such as inflammation, angiogenesis, and immune response, which may not be reflected in histological or clinical parameters [7, 8]. Third, serum proteins can be measured longitudinally, allowing for monitoring of disease progression and response to therapy over time. In high-risk individuals, such as hereditary predisposition carriers, serum biomarkers could provide early signs of developing cancer.

Several serum biomarkers have been investigated for their association with breast cancer survival. Carcinoembryonic antigen and CA15-3 are the most widely used serum protein biomarkers in metastatic breast cancer, but their prognostic value in early-stage breast cancer is limited [9, 10]. More modern techniques include the assessment of circulating tumor cells, DNA, RNA or microRNAs, but despite the rapid and promising development in these fields, there are still open issues regarding the technical optimization and standardization of these methods [11, 12].

Inflammation and the immune system are hallmarks of cancer, and they play a crucial role in both the early stages of breast carcinogenesis and breast cancer metastasis, especially in triple-negative and HER2-positive subtypes [13, 14]. The neutrophil-to-lymphocyte ratio, platelet-to-lymphocyte ratio and monocyte-to-lymphocyte ratio in the peripheral blood are all surrogates for systemic inflammation and promising prognostic factors in early-stage breast cancer but are still rarely used in clinical practice due to a lack of validation [15–17].

Due to the crucial role of inflammation and immunity in breast carcinogenesis and because of the lack of studies connecting these blood-assessed cancer hallmark proteins to prognosis, we measured preoperative serum levels of 92 immunity/inflammation-related proteins in 521 patients with early breast cancer followed in a prospective cohort and 61 patients with non-malignant breast conditions. Our aim was to develop an inflammation/immunity-related serum protein signature that could provide more accurate prognostic information than currently available tools.

## Methods

The patients who entered this prospective cohort were diagnosed in 2003–2013 with early-stage, invasive breast cancer. They received contemporary adjuvant treatments at the Oulu University Hospital (Table 1). Patients with a history of previous breast cancer or the presence of distant metastases at the time of diagnosis were excluded.

**Table 1:** Characteristics of the breast cancer patients in the discovery and validation cohorts

	Discovery cohort n (%)	Validation cohort n (%)	P-value^a^
Number of patients	413	108	
T class			0.211
T1	274 (66.3%)	67 (62.0%)	
T2	121 (29.3%)	40 (37.1%)	
T3	16 (3.9%)	1 (0.9%)	
T4	2 (0.5%)	0	
N class			0.518
N0	252 (61.0%)	67 (62.0%)	
N1	113 (27.4%)	27 (25.0%)	
N2	36 (8.7%)	13 (12.0%)	
N3	12 (2.9%)	1 (1.0%)	
Histopathology			0.372
Ductal	317 (76.8%)	81 (75.0%)	
Lobular	61 (14.8%)	21 (19.4%)	
Other	35 (8.5%)	6 (5.6%)	
Histopathological grade			0.462
Grade 1	72 (17.4%)	20 (18.5%)	
Grade 2	197 (47.7%)	58 (53.7%)	
Grade 3	127 (30.8%)	25 (23.1%)	
Unknown	17 (4.1%)	5 (4.6%)	
ER^b^ expression			0.008
0%	75 (18.6%)	7 (6.5%)	
1–9%	9 (2.2%)	5 (4.6%)	
10–59%	19 (4.6%)	3 (2.8%)	
> 59%	308 (74.6%)	92 (85.2%)	
Unknown	2 (0.5%)	1 (0.9%)	
PR expression			0.304
0%	107 (25.9%)	19 (17.6%)	
1–9%	57 (13.8%)	13 (12.0%)	
10–59%	44 (10.7%)	15 (13.9%)	
> 59%	202 (48.9%)	60 (55.6%)	
Unknown	3 (0.7%)	1 (0.9%)	
HER2 amplification			0.007
HER2 positive	51 (12.3%)	4 (3.7%)	
HER2 negative	362 (87.7%)	104 (96.3%)	
Ki-67 expression			0.959
< 5%	26 (6.3%)	7 (6.5%)	
5–14%	188 (45.5%)	54 (50%)	
15–30%	96 (23.3%)	24 (22.2%)	
> 30%	96 (23.3%)	22 (20.4%)	
Unknown	7 (1.6%)	1 (0.9%)	
Tumour type			1.000
Unifocal	324 (78.5%)	85 (78.7%)	
Multifocal	89 (21.5%)	23 (21.3%)	
Breast cancer subtypes			0.012
Luminal A-like	187 (45.3%)	57 (52.8%)	
Luminal B-like (HER2 negative)	119 (28.8%)	41 (37.9%)	
Luminal B-like (HER2 positive)	27 (6.5%)	2 (1.9%)	
HER2 positive, non-luminal	23 (5.6%)	2 (1.9%)	
Triple-negative	50 (12.1%)	5 (4.6%)	
Unknown	7 (1.7%)	1 (0.9%)	

^a^Fisher´s exact test

^b^ER, estrogen receptor; PR, progesterone receptor; HER2, human epidermal growth factor receptor-2

Serum samples were collected from all study participants on the day of their operation or the day before and were stored at -20 °C until use. While there were 555 early breast cancer patients with serum samples available, 34 patients did not pass the quality control in the proximity extension assay (PEA) analysis, resulting in 521 evaluable samples in the final cohort. For statistical analyses, the invasive cancers were randomly split into discovery (*n* = 413) and validation (*n* = 108) cohorts.

As controls, we used a retrospective cohort of 62 patients with atypical ductal hyperplasia or benign fibroadenoma from the same time interval who were to undergo breast surgery. As one serum sample did not pass the PEA quality control, the number of evaluable patients in this cohort was 61. These were split between the discovery (*n* = 42) and validation (*n* = 19) cohorts.

Histopathology was evaluated according to current WHO classifications, and tumor stage was assessed according to TNM classification [18]. The expressions of ER, PR and Ki-67 was assessed using immunohistochemistry (IHC), as previously described [19]. HER2 expression was assessed using IHC and chromogenic in situ hybridization (CISH) to confirm any positive results. Any sample with a positive result of six or more gene copies according to CISH was considered HER2 positive [20].

Tumors were classified into five intrinsic subtypes according to the ESMO Early Breast Cancer Clinical Practice Guidelines [21]. Luminal A-like carcinomas expressed both estrogen receptors (ER) and progesterone receptors (PR), Ki-67 was expressed in < 15% of their cells, and HER2 was not amplified. Luminal B-like (HER2 negative) carcinomas were also ER positive and HER2 negative, but they either showed Ki-67 expression in > 15% of their cells or were PR negative. Luminal B-like (HER2 positive) carcinomas still expressed ER, and they also overexpressed HER2. Triple-negative breast carcinomas (TNBC) were defined as tumors with no expression of ER, PR and HER2. HER2-positive (non-luminal) cases had HER2 amplification but no ER or PR expression.

### Proximity extension assay

Serum samples (25 µl) were analyzed for 92 proteins using an antibody-based proximity extension assay (PEA) (Olink Proteomics AB, Uppsala, Sweden) with the Olink Target Inflammation 96 library. Analyses were performed according to the manufacturer’s instructions at the University of Uppsala. PEA gives relative protein abundance levels in NPX (Normalized Protein eXpression) on log2 scale. Each assay has an experimentally determined lower limit of detection (LOD), which is defined as three standard deviations above background level. After excluding proteins with concentrations below the LOD in ≥ 75% of samples, data were available for 78 proteins (Additional file 1; Supplementary Table 1). The immuno-oncology panel was chosen a priori because of the known linkage between inflammation and the development of breast cancer.

### Statistical analyses

All data analyses were performed in the R environment (v. 4.2.2.). Median follow-up was estimated by the Reverse Kaplan-Meier method. Unsupervised clustering with Euclidean distance and ward.D linkage was carried out by the “pheatmap” package. For variable selection, we used the least absolute shrinkage and selection operator (LASSO) from the “glmnet” package (alpha = 1) with 10-fold cross-validation for model building in the discovery cohort and validated the model in the validation cohort [22]. Lambda value within one standard error of the minimum cross-validation error was used to select proteins for further analysis. The risk score was calculated for each sample as a linear sum of the levels of Lasso-selected proteins multiplied by their coefficients. The Wilcoxon rank sum test and Kruskal‒Wallis tests were used for non-parametric comparisons between two or more groups, respectively. Fisher’s exact test was used to assess whether differences in dichotomous clinical variables were significant between groups. The optimal cut-point for high vs. low signature groups was determined using the maximally selected rank statistics (maxstat package) in the discovery cohort and the same cut-off was used for the validation cohort.

Breast cancer -specific survival (BCSS) was calculated from the date of surgical tumor removal to the time of breast cancer-related death or the end of follow-up, while overall survival (OS) time ended at the time of any cause of death or the end of follow-up. Relapse-free survival (RFS) was calculated from the date of the operation to the date of the first confirmed local relapse in the ipsilateral or contralateral axilla, scar, or breast. Distant disease-free survival (DDFS) was calculated from the date of the operation to the date of the first confirmed distant relapse. Disease-free survival (DFS) combined both RFS and DDFS, with local and distant relapses as events.

In multivariable regression analyses, nodal status, tumor size, grade, and ER status were used as covariables with the 9-protein prognostic score. The Fine-Gray sub-distribution hazard model utilizing the “cmprsk” package was used to estimate the incidence of breast cancer -specific survival (BCSS), treating death resulting from causes other than breast cancer as a competing risk [23, 24]. Similarly, when assessing DDFS, DFS, and RFS, were deaths considered as competing risks. For OS analysis, Cox regression models were used. The “risksetROC” package was used to calculate the incident case/dynamic control receiving operator characteristics (ROC) curve as well as the area under the curve (AUC) across a range of time points (6, 12, 24, 36, 48, 60, 72, 120, and 168 months) for the clinical factors (tumor size, nodal status, grade, estrogen receptor) alone or in combination with the nine-protein signature according to the method described by Heagerty and Zheng and Song and Zhou [25, 26].

The study was approved by The Regional Ethics Committee of the Northern Ostrobothnia Hospital District (123/2016). The principles of the Declaration of Helsinki were followed. All patients signed informed consent before participation in the study.

## Results

### Patient characteristics

The median age of the 521 early-stage breast cancer patients included in the final cohort was 57 years [Interquartile range (IQR) 50–64 years], and the median follow-up time was 8.9 years (IQR 7.1–11.0). During the follow-up, there were 29 local recurrences (relapse in ipsilateral or contralateral axilla, scar or breast), 53 distant recurrences, 44 breast cancer-related deaths, and 74 deaths overall. Three hundred twelve (59.5%) patients received adjuvant chemotherapy, 452 (86.8%) received postoperative radiotherapy, and 343 (65.8%) received adjuvant endocrine therapy (Table 2). The patient and tumor characteristics were equally distributed between the discovery and validation cohorts, except for the tumor subtype and HER2 and ER status (Table 1).

**Table 2:** The distribution of adjuvant chemotherapy, radiotherapy and endocrine therapy among the early-stage breast cancer patients included into the analysis

	Discovery cohort n (%)	Validation cohort n (%)	P-value^a^
Number of patients	413	108	
Adjuvant chemotherapy			0.452
Anthracycline-based + taxane	93 (22.5%)	24 (22.2%)	
Anthracycline-based	82 (19.9%)	30 (27.8%)	
Other chemotherapy	18 (4.4%)	4 (3.7%)	
Trastuzumab + chemotherapy	51 (12.3%)	8 (7.4%)	
No adjuvant chemotherapy	167 (40.4%)	42 (38.9%)	
Missing	2 (0.5%)	0	
Adjuvant radiotherapy			0.425
Yes	361 (87.4%)	91 (84.3%)	
No	52 (12.6%)	17 (15.7%)	
The first prescribed adjuvant endocrine therapy			0.004
Tamoxifen	109 (26.4%)	39 (3.6%)	
Aromatase inhibitor	145 (35.1%)	45 (41.7%)	
Goserelin and tamoxifen	1 (0.2%)	1 (0.9%)	
Other endocrine therapy	2 (0.5%)	1 (0.1%)	
No endocrine therapy	154 (37.3%)	21 (19.4%)	
Missing	2 (0.5%)	1 (0.9%)	

^a^Fisher´s exact test

### Inflammatory protein landscape of breast cancer

**Fig. 1:**
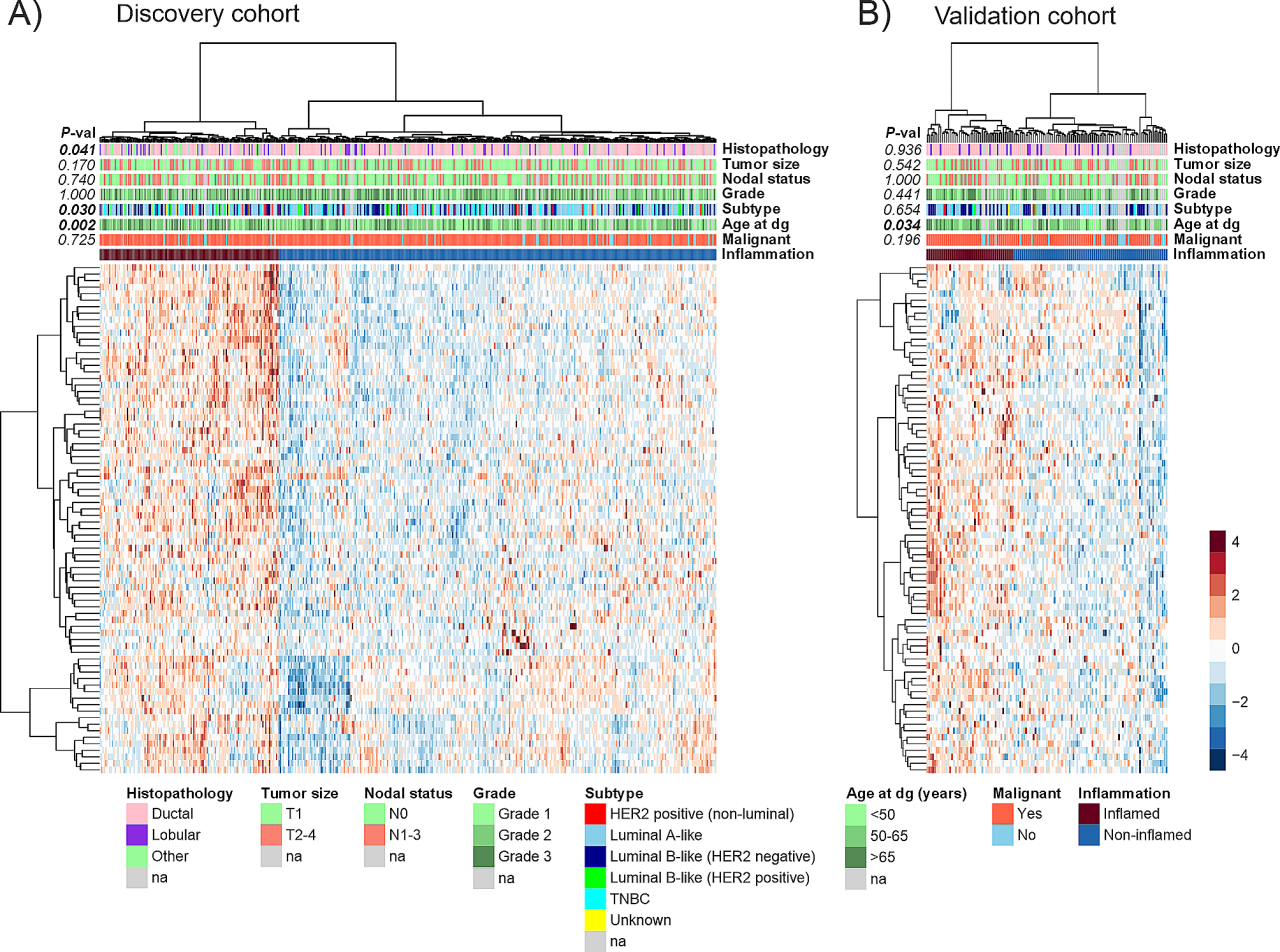
Serum inflammatory protein landscape in breast cancer. Heatmaps visualizing unsupervised hierarchical clustering of the immuno-oncological serum proteins in the discovery (**A**) and validation (**B**) cohorts. The data are Z score-normalized relative protein levels. Blue denotes lower protein levels, and reddish denotes higher protein levels. Rows represent proteins, and columns represent samples from breast cancer patients and nonmalignant controls. Clinical variables are annotated on the top of the heatmaps, and their distribution between the inflamed and non-inflamed clusters was assessed by Fisher’s exact test. Dg, diagnosis; na, not assigned; TNBC, triple-negative breast cancer

To obtain an overview of the inflammatory protein landscape of breast cancer, we first performed unsupervised hierarchical clustering analysis with the discovery cohort. In both the discovery and validation cohorts, the patients clustered in two major groups based on the levels of serum inflammatory proteins (Fig. 1A-B). The “inflamed group” with higher levels of the inflammatory proteins was significantly associated with older age in both cohorts (discovery, *P* = 0.002; validation *P* = 0.034) (Fig. 1A-B, Additional File 2; Supplementary Table 2). Of the other clinical characteristics, Luminal A subtype and ductal histologic subgroup were enriched among the “non-inflamed” group in the discovery cohort, but these data were not replicated in the validation cohort (Fig. 1A-B, Additional File 2; Supplementary Table 2).

### 9-protein risk score for predicting breast-cancer specific survival

**Fig. 2:**
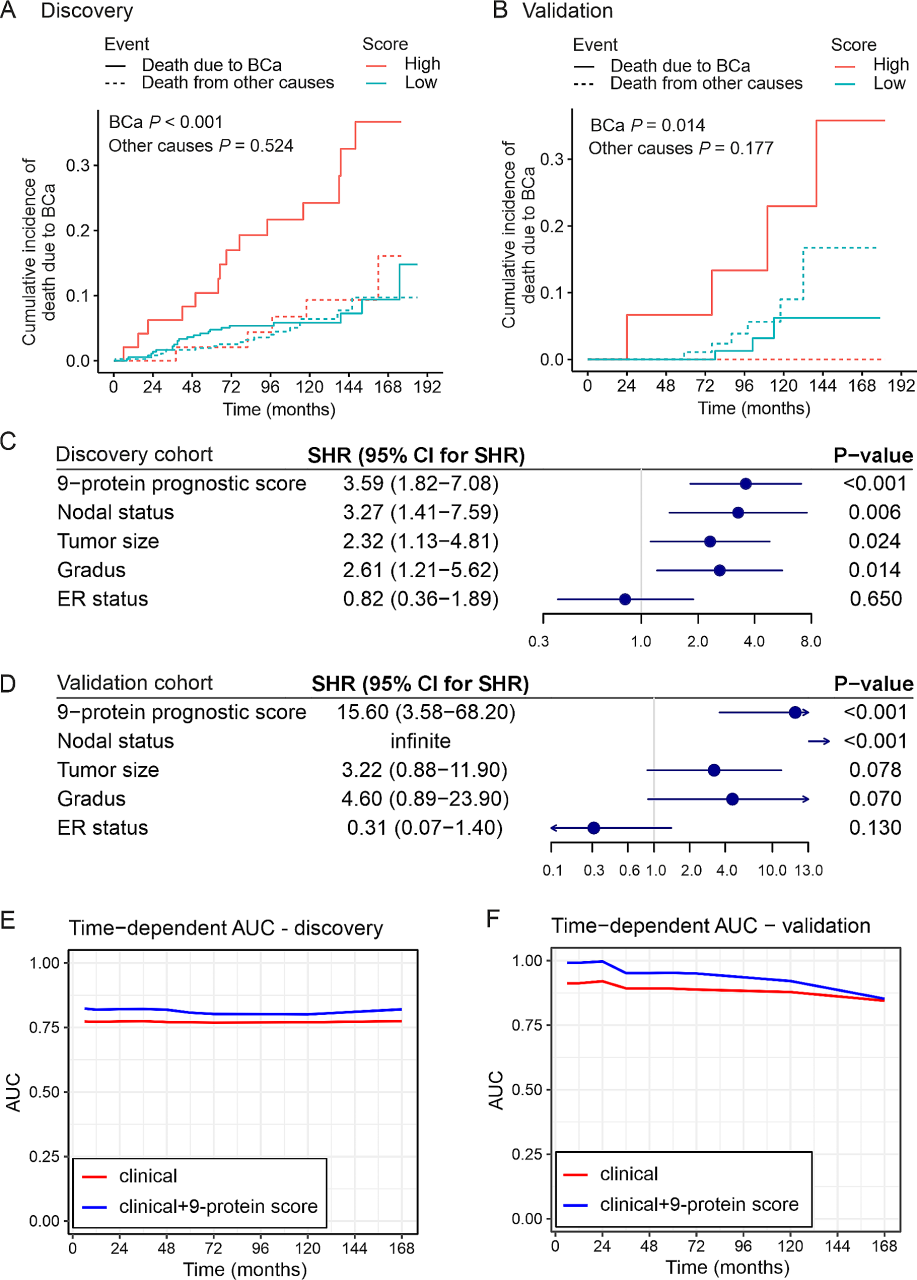
Association of the nine-protein prognostic score with breast cancer-specific survival. **A**-**B**) Cumulative incidence of death due to breast cancer according to the 9-protein prognostic score in the discovery (**A**) and validation (**B**) cohorts. Maximally selected rank statistics was used in the discovery cohort to determine the optimal cut-off of the score (high vs. low), and the same cut-off was used for the validation cohort. **C**-**D**) Multivariable regression analysis of breast cancer-specific survival in the discovery cohort (*n* = 413) and validation cohort (*n* = 108), including the nine-protein prognostic score and the most powerful traditional prognostic factors of early-stage breast cancer. In the models, death from causes other than breast cancer was treated as a competing risk. ER = estrogen receptor, SHR = subdistribution hazard ratio, CI = confidence interval E-F) Time-dependent receiver operating characteristic curves in the discovery (**E**) and validation (**F**) cohorts. The area under the curve (AUC) was higher in both cohorts at all timepoints when the nine-protein signature was added to the clinical factors (tumor size, nodal status, grade, estrogen receptor)

One of the main aims of the study was to develop a signature of immunity/inflammation-related proteins to predict especially breast cancer-specific survival. Variable selection with Lasso resulted in a 9-protein (CCL8, CCL23, CCL28, CSCL10, S100A12, IL-10, IL-10RB, STAMPB2 and TNFβ; Additional file 2; Supplementary Table 3) signature, which was associated with worse breast cancer-specific survival (SHR 9.56; 95% CI 3.17–28.8, *P* < 0.0001) as assessed with Fine-Gray competing risk model (Table 3). Cumulative incidence of death due to breast cancer was significantly increased in patients with high signature score in both the discovery and validation cohorts (*P* < 0.001 and *P* = 0.014, respectively) (Fig. 2A-B). The high signature score also predicted a dismal breast cancer-specific prognosis in a multivariable analysis in the discovery cohort (sub-distribution hazard ratio (SHR) 3.59; 95% confidence interval (CI) 1.82–7.08) (Fig. 2C). This was corroborated in the validation cohort (SHR 15.60, 95% CI 3.58–68.20) (Fig. 2D). In this model, the signature was a more dominant prognostic factor than the most powerful traditional prognostic factors of early-stage breast cancer. From the secondary endpoints, the signature predicted OS, DFS and DDFS, but not RFS in both the discovery and validation cohorts (Additional file 3; Supplementary Fig. 1A-B). The signature was distributed equally between the breast cancer biological subtypes (Additional file 3; Supplementary Fig. 2) and was not associated with ER, PR or HER2 (Additional file 3; Supplementary Fig. 3) or tumor size, nodal status or grade (Additional file 3; Supplementary Fig. 4). In the post-hoc analysis of node-negative or node-positive patients separately, the 9-protein score did not have statistically significant prognostic value among the node-negative patients (SHR 3.76, 95% CI 0.91–15.53), but only with the patients with node-positive breast cancer (SHR 3.51; 95% CI 1.60–7.67) (Additional file 2; Supplementary Table 4).

**Table 3:** The survival results of the studied endpoints from the univariable analysis

	Discovery cohort				Validation cohort		
	HR	95% CI	p-value		HR	95% CI	p-value
Breast cancer-specific survival	9.790	3.151–30.417	8.01*10^− 5^		28.002	1.804-434.579	0.017
Overall survival	7.150	2.938–17.397	1.45*10^− 5^		2.483	0.337–18.315	0.372
Distant disease-free survival	8.215	2.867–23.539	8.83*10^− 5^		9.425	1.077–82.505	0.043
Disease-free survival	4.701	1.924–11.486	0.001		6.375	1.110-36.615	0.038
Relapse-free survival	1.761	0.388–7.985	0.463		2.852	0.142–57.159	0.493

CI = Confidence Interval; HR = Hazard Ratio

In the time-dependent area under the receiver operating characteristic curve (AUC) analyses, the prognostic power of the model remained rather stable over time (Fig. 2D-E), although the number of cases was small at the end of the follow-up in the validation cohort.

### Signature to differentiate malignant from benign lesions

**Fig. 3:**
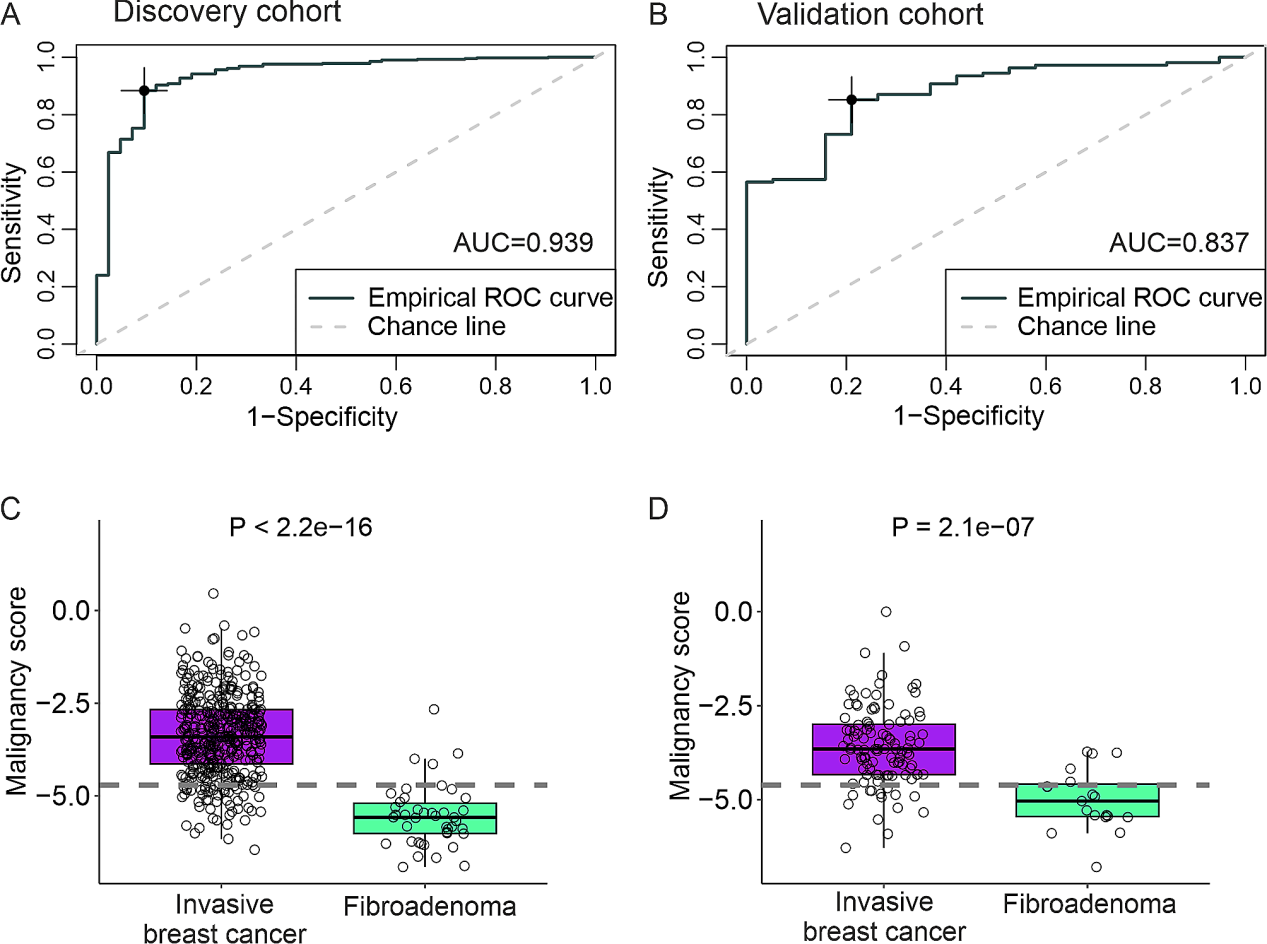
Protein signature for the classification of malignant breast cancer. **A**-**B**) Receiver operating characteristic (ROC) curves for the 17-protein malignancy score in the discovery (**A**) and validation (**B**) cohorts. The optimal Youden index point is marked in the plots (speficity and sensitivity 90% and 88%, respectively, in the discovery cohort, and 79% and 85% in the validation cohort). AUC, area under the curve. **C**-**D**) Box plots visualizing the distribution of the 17-protein malignancy score in invasive breast cancer samples and non-malignant fibroadenomas in the discovery (**C**) and validation (**D**) cohorts. The dashed line indicates the optimal cut-point (-4.7) of the score as determined by the ROC curve analysis

The overall inflammatory protein landscape did not separate malignant from benign lesions. However, using lasso variable selection, we constructed a 17-protein signature to differentiate malignant from benign lesions (Additional file 2; Supplementary Table 5). This signature consisted of, for instance, interleukins (IL-17 A, IL-6), chemokine ligands (CXCL1, CXCL5, CXCL9), and growth factors (CSF1, FGF19, VEGFA). Based on the levels of the signature proteins and their Lasso-coefficients, we calculated “a malignancy score”, which was predictive for the malignancy with excellent sensitivity and specificity in both the discovery and validation cohorts, and had a negative predictive value of 93% (Fig. 3A-D).

## Discussion

This long follow-up study with a prospectively collected cohort is the first to propose that immune/inflammation-related analysis of preoperative serum protein levels can (1) predict the survival of patients with early-stage breast cancer more accurately than established classical prognostic factors and (2) has the potential to distinguish benign from malignant breast lesions. As the here reported preoperative 9-protein signature was by far more accurate prognostic factor than tumor size, nodal status, histological grade or ER status, it has potential to be used in the individualization of adjuvant therapies, and surveillance.

The identified prognostic serum signature consisted of nine proteins with rather diverse immune and inflammatory functions, all with very little or no previous data on their prognostic value in early-stage breast cancer. IL-10R2 (also known as IL-10RB), which serves as the IL-22 receptor, received the most weight in the regression model as an indicator of poor prognosis and has not been studied earlier in clinical breast cancer materials at all. Another protein of the IL family included in the prognostic model was IL-10, a pleiotropic inflammatory and immune system regulator, which was only one of the nine identified proteins in the signature that predicted improved outcome [27]. Recently, high PEA-measured IL-10 levels were reported to be associated with worse progression-free survival in metastatic breast cancer [28]. In line with our results, high tissue expression of IL-10 was associated with improved DFS and BCSS in a series of 1380 early breast cancer patients in univariate analysis [29]. To the best of our knowledge, serum or plasma IL-10 levels have not been previously evaluated against prognosis in early-stage breast cancer.

Of the eight studied chemokine (C-C motif) ligands (CCL), CCL8, CCL23 and CCL28 were included in the nine-protein signature as risk-increasing variables. The identified CCL proteins have various carcinogenetic properties, such as the increase in breast cancer cell proliferation, chemoresistance development and T-cell and NK-cell regulation (CCL8), stimulation of angiogenesis and cancer cell proliferation (CCL23) and control of cell migration (CCL28) [30–33]. None of these proteins’ serum or plasma levels have been previously evaluated against the prognosis of early-stage breast cancer. Nevertheless, high breast cancer tissue CCL8 mRNA tissue levels have been reported to increase in breast cancers compared to adjacent healthy tissues and to be associated with worse RFS [32, 34]. In biliary tract cancers, elevated serum CCL23 predicted a dismal prognosis [35], and PEA-measured high plasma CCL28 levels implied worse survival in epithelial ovarian cancers [36]. Again, CCL28 levels in tumor tissue have a favorable prognostic role in luminal breast cancer but are associated with a worse prognosis in TNBC [37]. Based on their functions in biology and carcinogenesis, the current results imply that serum CCL8, CCL23 and CCL28 could also be drivers of aggressive breast cancer behavior in situ. Nevertheless, we did not have access to primary tumors to confirm this hypothesis, and it is also possible that the increased CCL levels could reflect an immunological response against primary tumor or subclinical metastases.

C-X-C motif ligand 10 (CXCL10) is an estrogen-regulated, proinflammatory cytokine that has been linked to the progression of several tumor types and was associated with poor outcome in the current study [38, 39]. Serum CXCL10 levels are increased in breast cancer patients compared to healthy controls and are related to endocrine therapy resistance in vitro [40, 41]. A small retrospective study suggested that serum CXCL10 levels alone may serve as a prognostic factor in breast cancer, although no validation cohort was available, and multivariate analysis was not performed [39].

The proteomics panel used in this study contained several proteins related to innate immunity, of which S100A12 (also known as EN-RAGE) and STAMBP were included in the nine-protein prognostic signature. Several members of the S100 proteins have been linked to breast cancer progression and metastasis [42, 43]. To the best of our knowledge, S100A12 has been specifically previously studied only in a single breast cancer publication, where the authors assessed the circulating S100A12 levels in 45 early-stage patients with ELISA and did not report any significant clinical findings [44]. Again, the gene expression of *S100A12* has been linked to worse outcomes in larger bladder, esophageal and gastric cancer materials [45–47]. In our study, S100A12 was strongly associated with DFS and DDFS endpoints, had higher serum levels in cancer compared to benign samples, and was also part of the 17-protein signature. The overexpression of *STAMBP* has been linked to metastasis formation in several solid cancers in vitro [47, 48]. Specifically, in breast cancer, *STAMBP* knockdown inhibited cell mobility and invasion by compromising EGFR/MAPK signaling pathway activation and inducing the degradation of actin-binding proteins [49].

Tumor necrosis factor-β (TNFβ, lymphotoxin-α) belongs to the TNF superfamily and is a potent activator of tumor cell proliferation, cell invasion, metastasis and inflammatory signals through stimulating the NF-κB pathway [50]. Consistent with these previous findings, TNFβ was identified as a part of the nine-protein risk signature in the current study. Although TNFβ gene polymorphisms have been rather broadly studied in breast cancer [51, 52], no study has previously evaluated TNFβ protein levels in tissue or bloodstream in relation to the survival of breast cancer patients.

Taken together, there is very sparse information available on the proteins included in the nine-protein signature in the previous literature regarding clinical breast cancer materials. Nevertheless, the results from other cancer types and preclinical breast cancer studies support the hypothesis that the levels of the identified proteins could play a role in breast cancer progression, not being solely signs of enhanced immune/inflammation response to primary tumor or subclinical metastasis. The identified signature performed similarly in all breast cancer subtypes, except for TNBC, which is known to have a diverse immunological environment from other subtypes and, on the other hand, comprised only 11% of the cohort [53].

There have been recent efforts to increase the diagnostic accuracy of mammography and breast ultrasound with blood proteomics in several prospective studies. Thus far, prospective data with validation results are available from the Mastocheck© and Videssa Breast© tests, which have reported sensitivities of 74.4% and 92.3% and specificities of 66.9% and 85.3% to separate malignant from benign lesions, respectively [54, 55]. In this context, the 17-protein signature identified here with a sensitivity of 88.4%, a specificity of 90.4% and negative predictive value of 93% performed at least equally as the published data.

We acknowledge some pitfalls in our study. None of our patients were treated with neoadjuvant chemotherapy, although this modality is increasing, especially in the node-positive HER2 + and TNBC subtypes. Otherwise, the adjuvant treatments used can be considered contemporary. The number of proteins finally included in the analysis was limited to 78, but the scope of the current study was to address whether inflammation/immunity-related proteomics could have an impact on survival, and the panel used can be considered sufficiently representative and versatile for this purpose. We had no access to tumor tissues, and the comparison of serum protein levels with those in primary tumors and with tumor-infiltrating lymphocytes could have been able to provide more mechanistic insights into the current results. Additionally, we did not have external validation cohorts in our study, although this was compensated using internal validation. Again, the cohort included a relatively large number of patients with sufficient follow-up and comprehensive clinical and pathological data. As we used a prospective, non-selected cohort of patients, the number ER-negative and HER2-positive subtypes were low, especially in the validation cohort, and other, subtype-specific cohorts are required to confirm the results specifically in each subtype. Nevertheless, there were no signs of the diverse distribution of the proteins between the subtypes. Finally, despite the 92 proteins included in the panel were selected to cover the most essential inflammatory-related pathways, also other physiological and pathological processes than inflammation can affect to their expression.

Since up to 19% of patients in the high-risk signature suffered a distant relapse during the follow-up (compared to 5% in the low-risk group), the high-risk patients could in theory benefit from more intense surveillance and even from the more intensive adjuvant treatments. Based on the current results, there could also be potential for de-escalation studies for patients belonging to the low-risk group. Again, the 17-protein signature could offer ground for future studies, with the potential to validate non-invasive and inexpensive liquid biopsy-based screening, e.g., in patients with a hereditary predisposition for breast cancer. Taken together, this is the first large-scale study in which we discovered an inflammatory serum protein signature that reliably predicts survival in patients with primary breast cancer. We expect growing interest to further explore this novel minimally invasive biomarker in the near future.

### Electronic supplementary material

Below is the link to the electronic supplementary material.

Supplementary Material 1

Supplementary Material 2

Supplementary Material 3

## Data Availability

The data generated in this study are available upon request from the corresponding author.
