# Supplementary material for: Serum protein profiling reveals an inflammation signature as a predictor of early breast cancer survival
Source: Breast Cancer Res. 2024 Apr 9;26:61. doi: 10.1186/s13058-024-01812-x (PMC11005292; doi:10.1186/s13058-024-01812-x)
Supplement: Supplementary file 2 — Supplementary Material 2 [file 13058_2024_1812_MOESM2_ESM.docx]

**Additional File 2: Supplementary Tables 2-4**

**Supplementary Table 2. Distribution of the patients’ clinical and pathological characteristics in the inflamed and non-inflamed subgroups.**

|  | **Discovery cohort** | | | **Validation cohort** | | |
| --- | --- | --- | --- | --- | --- | --- |
|  | **Non-inflamed** | **Inflamed** | **P-value^a^** | **Non-inflamed** | **Inflamed** | **P-value** |
| Number of patients | 292 | 121 |  | 66 | 42 |  |
| Age years [median (IQR^b^)] | 56 (49-62) | 61 (53-68) | 0.002 | 56 (49-63) | 60 (52-70) | 0.034 |
| <50 years | 84 (29) | 18 (15) |  | 18 (27) | 5 (12) |  |
| 50-65 years | 151 (52) | 64 (53) |  | 36 (55) | 21 (50) |  |
| >65 years | 57 (19) | 39 (32) |  | 12 (18) | 16 (38) |  |
| Tumor size |  |  | 0.170 |  |  | 0.542 |
| T1 | 200 (68) | 74 (61) |  | 39 (59) | 28 (67) |  |
| T2-4 | 92 (32) | 47 (39) |  | 27 (41) | 14 (33) |  |
| Nodal status |  |  | 0.740 |  |  | 1.000 |
| N0 | 180 (62) | 72 (60) |  | 41 (62) | 26 (62) |  |
| N1-3 | 112 (38) | 49 (40) |  | 25 (38) | 16 (38) |  |
| Histopathology |  |  | 0.041 |  |  | 0.936 |
| Ductal | 233 (80) | 84 (70) |  | 50 (76) | 31 (74) |  |
| Lobular | 40 (14) | 21 (17) |  | 12 (18) | 9 (21) |  |
| Other | 19 (6) | 16 (13) |  | 4 (6) | 2 (5) |  |
| Histopathological grade |  |  | 1.000 |  |  | 0.441 |
| Grade 1 | 54 (19) | 18 (15) |  | 11 (16) | 9 (21) |  |
| Grade 2 | 138 (47) | 59 (49) |  | 38 (58) | 20 (48) |  |
| Grade 3 | 91 (31) | 36 (30) |  | 13 (20) | 12 (29) |  |
| Unknown | 9 (3) | 8 (6) |  | 4 (6) | 1 (2) |  |
| Breast cancer subtypes |  |  | 0.030 |  |  | 0.654 |
| Luminal A-like | 142 (49) | 45 (37) |  | 38 (58) | 19 (45) |  |
| Luminal B-like (HER2 negative) | 81 (28) | 38 (31) |  | 23 (35) | 18 (43) |  |
| Luminal B-like (HER2 positive) | 19 (6) | 8 (7) |  | 1 (1.5) | 1 (2) |  |
| HER2 positive, non-luminal | 15 (5) | 8 (7) |  | 1 (1.5) | 1 (2) |  |
| Triple-negative | 34 (12) | 16 (13) |  | 3 (4) | 2 (5) |  |
| Unknown | 1 (0.3) | 12 (5) |  | 0 | 1 (2) |  |

^a^Fisher’s exact test

^b^IQR; Interquartile range

**Supplementary Table 3. The proteins included in the prognostic signature and their coefficients in the model.**

| Protein | Coefficient |
| --- | --- |
| CCL8 | 0.135 |
| CCL23 | 0.114 |
| CCL28 | 0.04937 |
| CXCL10 | 0.00957 |
| S100A12 (EN-RAGE) | 0.0318 |
| IL-10 | -0.0372 |
| IL10-RB | 0.256 |
| STAMBP | 0.0747 |
| TNFβ | 0.00747 |

**Supplementary Table 4. Multivariable analyses separately in N0 and N1 patients in the discovery cohort.**

N0 patients (n=252)

|  | SHR | 95% CI | P-value |
| --- | --- | --- | --- |
| 9-protein score | 3.76 | (0.91-15.53) | 0.067 |
| Tumor size | 5.45 | (1.44-20.55) | 0.012 |
| Grade | 3.57 | (0.65-19.54) | 0.140 |
| ER status | 0.83 | (0.12-5.65) | 0.840 |

N+ patients (n=161)

|  | SHR | 95% CI | P-value |
| --- | --- | --- | --- |
| 9-protein score | 3.51 | (1.60-7.67) | 0.002 |
| Tumor size | 1.69 | (0.78-3.68) | 0.180 |
| Grade | 2.30 | (0.99-5.28) | 0.051 |
| ER status | 0.83 | (0.33-2.09) | 0.690 |

SHR=sub-distribution hazard ratio; CI=Confidence interval

**Supplementary Table 5. The proteins included in the signature differentiating benign from the malignant breast lesions and their coefficients in the model.**

| **Protein** | **Coefficient** |
| --- | --- |
| 4E-BP1 | -0.13147 |
| ADA | -0.75332 |
| CASP-8 | 1.625627 |
| CD8A | -0.17454 |
| CSF-1 | -0.77899 |
| CXCL1 | -0.10202 |
| CXCL5 | -0.17023 |
| CXCL9 | 0.183491 |
| EN-RAGE | 0.058888 |
| FGF-19 | 0.131862 |
| Flt3-L | 0.235598 |
| IL-17A | -0.27445 |
| IL-6 | -0.0118 |
| LIF-R | 0.468501 |
| OSM | -0.53576 |
| SIRT2 | -0.51103 |
| VEGFA | -0.07033 |
