# Supplementary material for: Serum protein profiling reveals an inflammation signature as a predictor of early breast cancer survival
Source: Breast Cancer Res. 2024 Apr 9;26:61. doi: 10.1186/s13058-024-01812-x (PMC11005292; doi:10.1186/s13058-024-01812-x)
Supplement: Supplementary file 3 — Supplementary Material 3 [file 13058_2024_1812_MOESM3_ESM.docx]

Additional File 3: Supplementary Figures 1-4


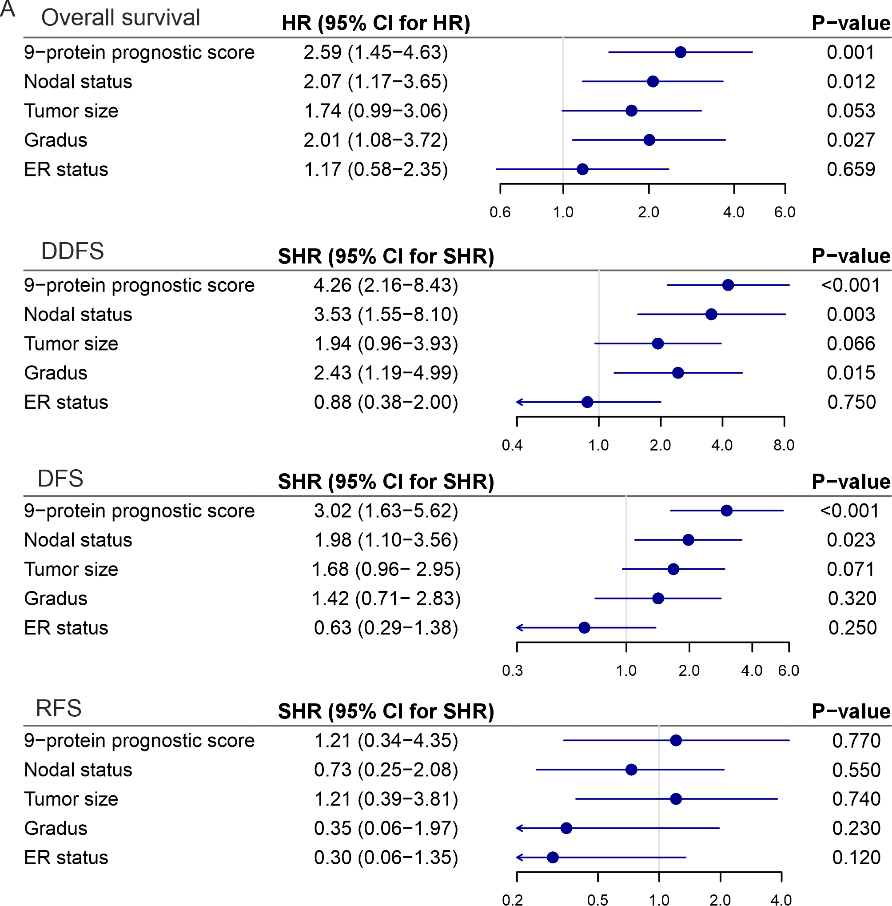


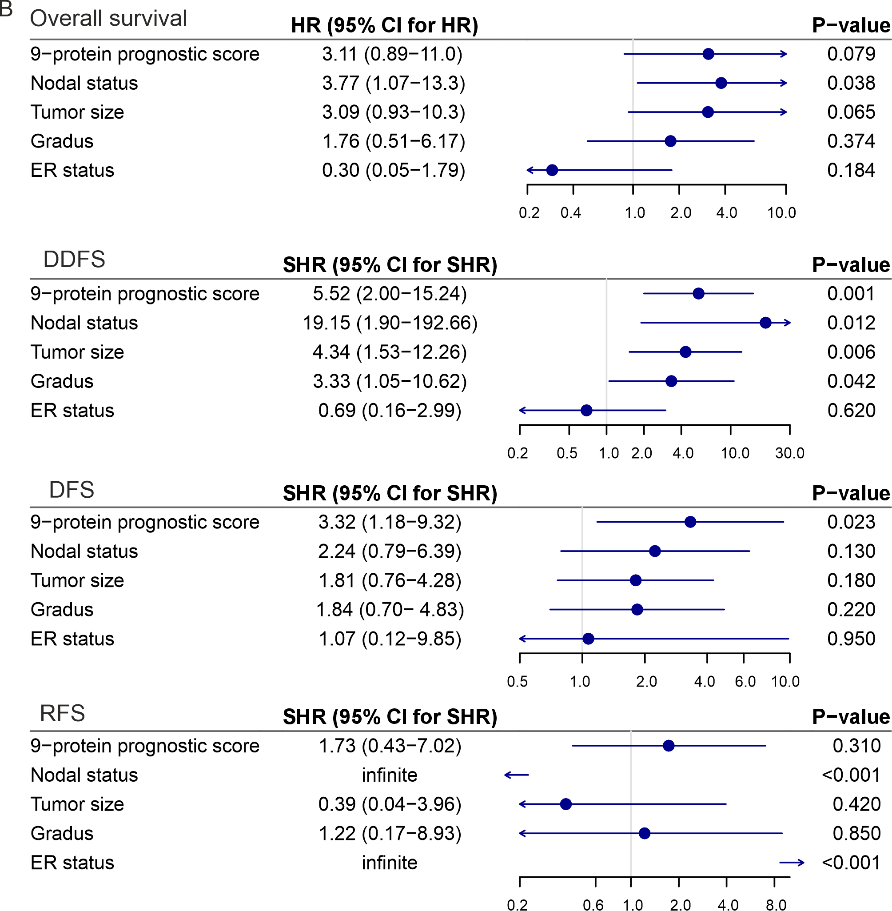


**Supplementary Figure 1.** Multivariable analysis of the 9-protein prognostic score with the most powerful traditional prognostic factors of early-stage breast cancer. A-B) Forest plots show the data for overall survival (OS), distant disease-free survival (DDFS), disease-free survival (DFS), and relapse-free survival (RFS) in the discovery (A) and validation (B) cohorts. HR, Hazard ratio; CI, Confidence interval; SHR, sub-distribution hazard ratio; ER, estrogen receptor.


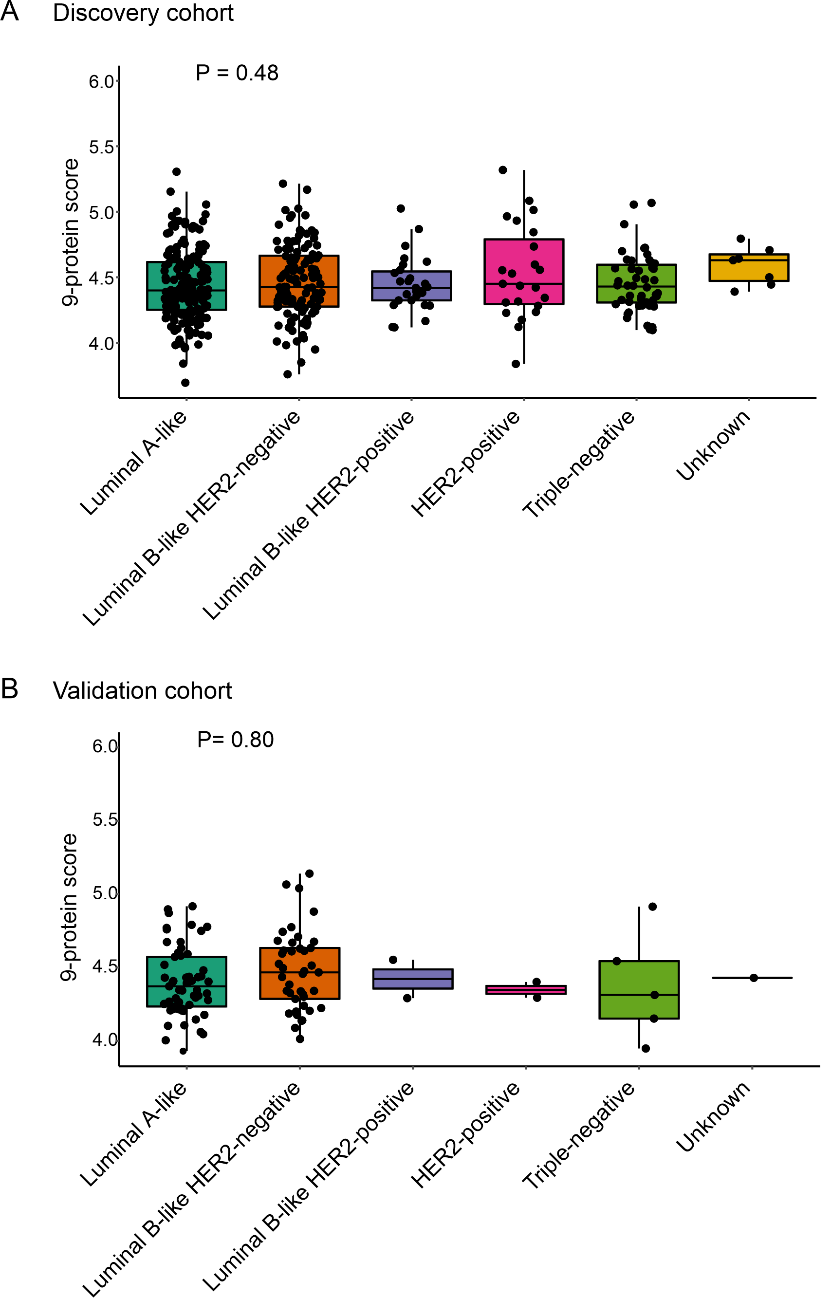


**Supplementary Figure 2.** Distribution of the 9-protein prognostic score between breast cancer subtypes in the discovery (A) and validation (B) cohorts.


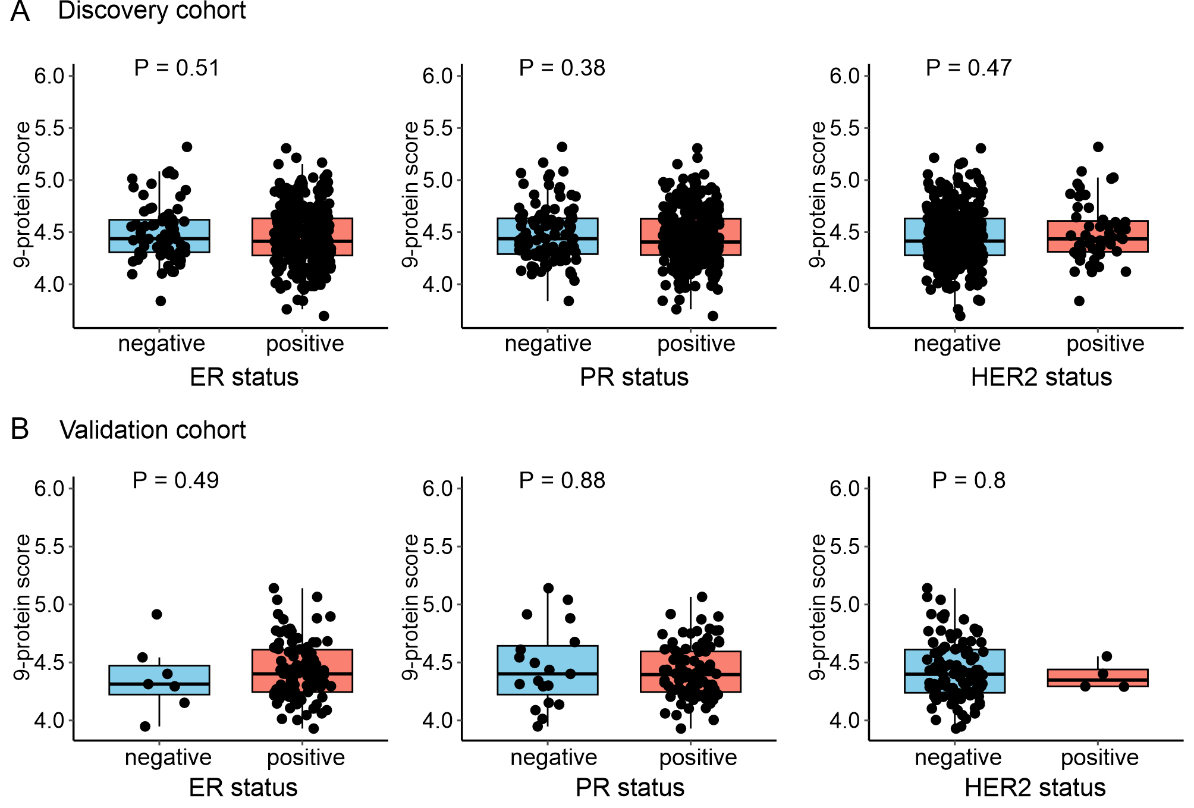


**Supplementary Figure 3.** Distribution of the 9-protein prognostic score according to the estrogen receptor (ER), progesterone receptor (PR) and human epidermal growth receptor-2 (HER2) status in the discovery (A) and validation (B) cohorts.


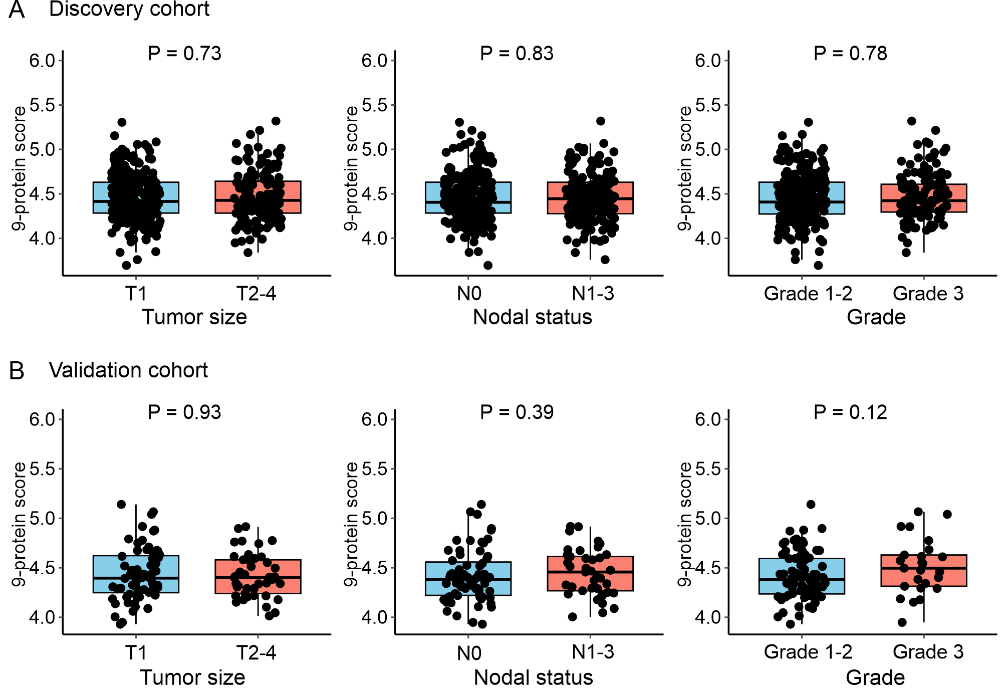


**Supplementary Figure 4.** Distribution of the 9-protein prognostic score according to the tumor size, nodal status, and grade in the discovery (A) and validation (B) cohorts.
